# Retraction Note: The Hopewell airburst event, 1699–1567 years ago (252–383 CE)

**DOI:** 10.1038/s41598-023-41237-8

**Published:** 2023-08-30

**Authors:** Kenneth Barnett Tankersley, Stephen D. Meyers, Stephanie A. Meyers, James A. Jordan, Louis Herzner, David L. Lentz, Dylan Zedaker

**Affiliations:** 1 Department of Anthropology, Department of Geology, University of Cincinnati, Cincinnati, OH 45221 USA; 2 Department of Geology, University of Cincinnati, Cincinnati, OH 45221 USA; 3 Department of Biology, University of Cincinnati, Cincinnati, OH 45221 USA; 4 Reston Stable Isotope Laboratory, United States Geological Survey, Reston, VA 20192 USA

Retraction of: *Scientific Reports* 10.1038/s41598-022-05758-y, published online 01 February 2022

The Editors have retracted this Article.

Following publication of this Article concerns have been raised by Nolan et al.^1^ about errors in methodology, analyses, and interpretation of the data in the Article, indicating that the study does not provide data to support the claims of an airburst event or that such an event led to the decline of the Hopewell culture. Given these concerns, the Editors no longer have confidence that the conclusions presented are adequately supported.

James A. Jordan agrees with this retraction. Kenneth Barnett Tankersley, Stephen D. Meyers, Stephanie A. Meyers, Louis Herzner, David L. Lentz and Dylan Zedaker did not respond to correspondence from the Editors about this retraction.
